# Supplementary material for: Novel pedigree analysis implicates DNA repair and chromatin remodeling in multiple myeloma risk
Source: PLoS Genet. 2018 Feb 1;14(2):e1007111. doi: 10.1371/journal.pgen.1007111 (PMC5794067; doi:10.1371/journal.pgen.1007111)
Supplement: S1 Table — (PDF) [file pgen.1007111.s004.pdf]

S1 Table. Genome-wide thresholds and segments.

Pedigree	Total MM	Genotyped MM	Meioses	Subsets	Total SGSs	Total SGSs >= 20 Markers	Optimized SGSs	Significant	Suggestive
UT 260	31	3	16	4	2,512,103	2,050,195	10,321	$6.20x10^{-6}$	$1.19x10^{-4}$
UT 2122	5	3	18	4	2,510,600	2,045,974	10,369	$5.05x10^{-6}$	$1.03x10^{-4}$
UT 4823	4	3	13	4	2,513,732	2,055,027	10,091	$1.35x10^{-5}$	$1.98x10^{-4}$
UT 20245	4	3	13	4	3,153,413	2,605,542	10,057	$1.40x10^{-5}$	$2.05x10^{-4}$
UT 34955	12	3	16	4	2,511,001	2,044,970	10,299	$5.66x10^{-6}$	$1.11x10^{-4}$
UT 48833	20	4	23	11	6,764,481	5,158,345	9,942	$6.81x10^{-7}$	$1.42x10^{-5}$
UT 546699	14	2	11	1	638,525	549,470	6,697	$1.10x10^{-4}$	$1.17x10^{-3}$
UT 549917	18	4	21	11	6,762,838	5,148,182	9,534	$8.33x10^{-7}$	$1.64x10^{-5}$
UT 571744	37	3	20	4	2,513,408	2,053,132	10,260	$3.78x10^{-6}$	$8.50x10^{-5}$
UT 576834	9	4	16	11	6,765,500	5,157,953	9,857	$3.53x10^{-6}$	$4.63x10^{-5}$
UT 651626	6	3	13	4	2,514,522	2,056,852	9,961	$1.18x10^{-5}$	$1.82x10^{-4}$
