# Supplementary material for: Novel pedigree analysis implicates DNA repair and chromatin remodeling in multiple myeloma risk
Source: PLoS Genet. 2018 Feb 1;14(2):e1007111. doi: 10.1371/journal.pgen.1007111 (PMC5794067; doi:10.1371/journal.pgen.1007111)
Supplement: S2 Table — Total MM, MGUS, and controls in each pedigree and from each site. (PDF) [file pgen.1007111.s005.pdf]

S2 Table. Whole-exome sequenced families. Total MM, MGUS, and controls in each pedigree and from each site.

FAMILY	MM	MGUS	CONTROL
**International Agency for Research on Cancer, France**	**8**	**0**	**0**
IARC-unknown-2	2	0	0
IARC-unknown-1	3	0	0
IARC-unknown-3	3	0	0
**INSERM, France**	**36**	**38**	**0**
DEL-Lyon-0208	1	1	0
FRA-Amiens-1008	1	1	0
LAV-Reims-0609	1	1	0
PER-Paris-0309	1	1	0
RAN-Blois-0208	1	1	0
SOU-Paris-0809	1	1	0
WOE-Dunkerque-0109	1	1	0
DAB-Rennes-1109	1	2	0
DER-Lyon-0608	1	2	0
HIN-Rennes-1109	1	2	0
LEC-Reness-0608	1	2	0
MIO-Brest-1112	1	2	0
PET-Nice-0909	1	2	0
FOU-Chartres-1110	1	3	0
SER-Toulouse-0411	1	3	0
BAE-Nice-1110	2	0	0
BER-Nancy-0609	2	1	0
GRA-Tours-1013	2	1	0
NAV-Abbeville-1109	2	1	0
ROU-Paris-0308	2	1	0
GIR-Puy en Velay-0312	2	2	0
MAR-Lyon-0907	2	2	0
RON-Valence-1109	2	2	0
SIN-Lille-0108	2	2	0
DEB-Nancy-0609	3	1	0

FAMILY	MM	MGUS	CONTROL
**Mayo Clinic, Minnesota**	**10**	**8**	**10**
446	1	1	0
476	1	1	0
500	1	1	0
447	1	1	1
458	1	1	2
466	1	1	2
472	1	1	2
487	1	1	2
316	2	0	1
**Memorial Sloan Kettering Cancer Center, New York**	**14**	**0**	**0**
fm4532	2	0	0
fm5066	2	0	0
fm5329	2	0	0
fm5815	2	0	0
fm5885	2	0	0
fm4493	4	0	0
**Weill Cornell Medical College, New York**	**2**	**0**	**0**
MM12	2	0	0
**University of Utah School of Medicine, Utah**	**28**	**0**	**0**
48833	4	0	0
549917	4	0	0
571744	3	0	0
2122	3	0	0
260	3	0	0
34955	3	0	0
576834	4	0	0
4823	3	0	0
20245	3	0	0
651626	3	0	0
546699	3	0	0

Note: Some of MM cases in the Utah pedigrees fall in more than one high-risk pedigree.
