# Supplementary material for: Novel pedigree analysis implicates DNA repair and chromatin remodeling in multiple myeloma risk
Source: PLoS Genet. 2018 Feb 1;14(2):e1007111. doi: 10.1371/journal.pgen.1007111 (PMC5794067; doi:10.1371/journal.pgen.1007111)
Supplement: S3 Table — Burden testing results (based on 1,063 MM/MGUS cases and 964 unaffected controls), SGS and prioritized SNV results, and intolerance to missense and loss of function variants (based on ExAC population data). (PDF) [file pgen.1007111.s006.pdf]

S3 Table. GG-NER Incision Complex genes. Burden testing results (based on 1063 MM/MGUS cases and 964 unaffected controls), SGS and prioritized SNV results, and intolerance to missense and loss of function variants (based on ExAC population data).

Gene	Position	Burden (p-value)	SGS	SNV	Intolerance to MS (Z)	Intolerance to LoF (pLI)
USP45	6:99880182-99963565	0.711	significant	Y	-0.74	0.00
CHD1L	1:146714291-146767447	0.006^{+}			-1.82	0.00
PARP1	1:226548392-226595801	0.036^{+}			0.57	0.01
RPA2	1:28218049-28241236	0.019^{+}			0.41	0.89
ERCC3	2:128014866-128051752	0.020^{+}		Y	1.17	0.00
GTF2H2	5:70330951-70363497	0.005^{+}			2.27	0.72
GTF2H5	6:158589379-158620376	0.532			-0.70	0.05
GTF2H4	6:30875977-30882814	0.930			0.74	0.26
RPA3	7:7676575-7758238	1.000			-0.73	0.00
XPA	9:100437191-100459691	0.104			0.52	0.00
GTF2H1	11:18343816-18388590	0.001‡			0.59	1.00
DDB2	11:47236493-47260769	0.929			1.25	0.01
DDB1	11:61066919-61100684	0.001‡			5.67	1.00
GTF2H3	12:124118286-124147151	0.957			0.04	0.00
ERCC5	13:103504468-103524748	0.462			-0.61	0.00
CUL4A	13:113863931-113919392	0.390			2.73	1.00
PARP2	14:20811773-20826063	0.190			-0.55	0.00
ERCC4	16:14014014-14046205	0.371		Y	-1.06	0.00
RPA1	17:1733273-1802848	0.480			0.43	0.31
ERCC2	19:45854649-45873845	0.503	suggestive		0.44	0.00
ERCC1	19:45910591-45927177	0.402	suggestive		-0.13	0.00
RBX1	22:41347351-41369019	-			2.68	0.94
CUL4B	X:119658446-119709684	-	-		3.88	1.00

Legend: Position – build HG19; Burden – p-values based on the c-alpha test of high-impact variants with AAF < 0.001 (see Methods section), "-" indicates gene not tested (no variants observed), ‡significant after multiple testing correction p < 0.0022 (=0.05/23), $^{+}$nominally significant p < 0.05; SGS – gene captured by a genome-wide significant or suggestive shared genomic segment, "-" indicates not tested (SGS only looks at autosomal chromosomes); SNV – "Y" indicates a single nucleotide variant with AAF < 0.001, high or moderate deleteriousness, and observed segregating in a high-risk MM pedigree or pathogenic in ClinVar; Intolerance to MS – gene's intolerance to missense variants based on analysis of ExAC data[41], signed Z score based on deviation of observed counts from expected, positive Z indicates intolerance to variation; LoF – based on analysis of ExAC data[41], Loss of Function (LoF) variants include splice donor or acceptor or non-sense SNVs, genes with a probability of LoF Intolerance (pLI) >= 0.9 are considered extremely intolerant to LoF SNVs.
