# Supplementary material for: Novel pedigree analysis implicates DNA repair and chromatin remodeling in multiple myeloma risk
Source: PLoS Genet. 2018 Feb 1;14(2):e1007111. doi: 10.1371/journal.pgen.1007111 (PMC5794067; doi:10.1371/journal.pgen.1007111)
Supplement: S4 Table — (PDF) [file pgen.1007111.s007.pdf]

S4 Table. Evidence for endonuclease regulation of DNA repair.

Gene	Cases	Me	Family	Type	Chr	Position	Len	P-Value	dbSNP	Conseq	Impact	ClinVar	AAF
USP45	3	20	Utah 571744	SGS	6	98,489,655—100,243,996	1.8	$3.3x10^{-6‡}$					
USP45	3(2)	3	PET-Nice 0909	SNV	6	99,891,443			None	p.Gln691*	SG		None
USP45	2(1)	2	Mayo 458	SNV	6	99,893,787			None	p.Gln621Glu	MS		None
ERCC1 *ERCC2*	3	16	Utah 34955	SGS	19	45,716,198—46,509,578	0.8	$6.6x10^{-5+}$					
ERCC4	1	0		SNV	16	14,041,848			rs121913049	p.Arg799Trp	MS	Pathogenic	0.0008
ERCC3	1	0		SNV	2	128,036,759			rs768687646	p.Arg574Ter	SG	Same domain as pathogenic SNV	0.0000

Legend: Cases – number of MM and MGUS cases (number of MGUS) with genotype or exome DNASeq data who share the SGS region or carry the SNV; Me – meioses between cases; Type – SGS: shared genomic segment, SNV: single nucleotide variant; Position – build HG19; Len – length in mega-bases; p-value – SGS p-value (significant and suggestive genome-wide thresholds were $3.8x10^{-6}$ and $8.5x10^{-5}$ for Utah 571744 and $5.7x10^{-6}$ and $1.1x10^{-4}$ for Utah 34955), ‡genome-wide significant, +genome-wide suggestive; Conseq – exome-variant consequence; Impact – SG: stop gain variant, MS: missense variant; AAF – alternate allele frequency based on the non-TCGA, non-Finnish, European gnomAD individuals, “None” indicates the region had good coverage in gnomAD, but the variant has not been observed in gnomAD, “AAF = 0” indicates the variant has been observed in another ethnicity in gnomAD.
