# Supplementary material for: Novel pedigree analysis implicates DNA repair and chromatin remodeling in multiple myeloma risk
Source: PLoS Genet. 2018 Feb 1;14(2):e1007111. doi: 10.1371/journal.pgen.1007111 (PMC5794067; doi:10.1371/journal.pgen.1007111)
Supplement: S5 Table — Burden testing results (based on 1,063 MM/MGUS cases and 964 unaffected controls), SGS and prioritized SNV results, and intolerance to missense and loss of function variants (based on ExAC population data). (PDF) [file pgen.1007111.s008.pdf]

S5 Table. SWI/SNF Complex genes. Burden testing results (based on 1063 MM/MGUS cases and 964 unaffected controls), SGS and prioritized SNV results, and tolerance to missense and loss of function variants (based on ExAC population data).

Gene	Position	Burden (p-value)	SGS	SNV	Intolerance to MS (Z)	Intolerance to LoF (pLI)
ARID1A	1:27022522-27108601	0.001‡	2 pedigrees, borderline suggestive	Y	4.10	1.00
ACTL6A	3:179280708-179306193	0.001‡			2.93	0.99
SMARCC1	3:47627378-47823405	0.821			2.39	1.00
PBRM1	3:52579368-52713739	0.505	borderline suggestive		3.48	1.00
ARID1B	6:157099064-157531913	0.002‡			3.39	1.00
ACTL6B	7:100240726-100254084	0.187			4.04	0.99
SMARCD3	7:150936059-150945749	0.001‡			3.16	0.23
SMARCA2	9:2015342-2193623	0.334			5.57	1.00
ARID2	12:46123620-46301819	0.045+			1.87	1.00
SMARCD1	12:50478983-50494494	0.458			3.95	1.00
SMARCC2	12:56555636-56583351	0.001‡			4.26	1.00
SMARCE1	17:38783976-38804103	0.001‡			2.96	1.00
SMARCD2	17:61909441-61920351	0.685			2.37	0.98
SMARCA4	19:11071598-11172958	0.001‡			8.36	1.00
SMARCB1	22:24129150-24176705	-			4.51	1.00

Legend: Position – build HG19; Burden – p-values based on the c-alpha test of high-impact variants with AAF < 0.001 (see Methods section), "-" indicates gene not tested (no variants observed), ‡significant after multiple testing correction p < 0.0033 (=0.05/15), +nominally significant p < 0.05; SGS – gene captured by a border-line suggestive shared genomic segment; SNV – single nucleotide variant with AAF < 0.001, high or moderate deleteriousness, and observed segregating in a high-risk MM pedigree or pathogenic in ClinVar; Intolerance to MS – the gene's intolerance to missense variants based on analysis of ExAC data[41], signed Z score based on deviation of observed counts from expected, positive Z indicates intolerance to variation; Intolerance to LoF – based on analysis of ExAC data[41], Loss of Function (LoF) variants include splice donor or acceptor or non-sense SNVs, genes with a probability of LoF Intolerance (pLI) >= 0.9 are considered extremely intolerant to LoF SNVs.
