# Supplementary material for: Novel pedigree analysis implicates DNA repair and chromatin remodeling in multiple myeloma risk
Source: PLoS Genet. 2018 Feb 1;14(2):e1007111. doi: 10.1371/journal.pgen.1007111 (PMC5794067; doi:10.1371/journal.pgen.1007111)
Supplement: S6 Table — (PDF) [file pgen.1007111.s009.pdf]

S6 Table. Evidence for SWI/SNF chromatin remodeling.

Gene	MM	Me	Family	Type	Chr	Position	Len	P-Value	dbSNP	Conseq	Impact	AAF
ARID1A	3	12	Utah 576834	SGS	1	24,389,214—33,298,821	8.9	$3.0x10^{-4}$				
ARID1A	3	16	Utah 260	SGS	1	26,224,634—27,384,988	1.2	$2.1x10^{-4}$				
ARID1A	3	12	Utah 576834	SNV	1	27,023,162			rs752026201	p.Ser90Gly	MS	0.0002
ARID1A	2	4	Cornell MM12	SNV	1	27,089,712			rs140664170	p.Met890Val	MS	0.0001
PBRM1	4	21	Utah 549917	SGS	3	52,013,884—53,556,222	1.5	$2.2x10^{-5}$				

Legend: MM – number of MM cases (with genotype or exome DNASeq data) in the family who share the SGS region or carry the SNV; Me – meioses between MM cases; Type – SGS: shared genomic segment, SNV: single nucleotide variant; Position – build HG19; Len – length in mega-bases; p-value – SGS p-value (significant and suggestive genome-wide thresholds were $3.5x10^{-6}$ and $4.6x10^{-5}$ for Utah 576834, $6.2x10^{-6}$ and $1.2x10^{-4}$ for Utah 260, and $8.3x10^{-7}$ and $1.6x10^{-5}$ for Utah 549917); Conseq – exome-variant consequence; Impact – MS: missense variant; AAF – alternate allele frequency based on the non-TCGA, non-Finnish, European gnomAD individuals.
